# Supplementary material for: Psychosocial health of school-going adolescents during the COVID-19 pandemic: Findings from a nationwide survey in Bangladesh
Source: PLoS One. 2023 Mar 27;18(3):e0283374. doi: 10.1371/journal.pone.0283374 (PMC10042372; doi:10.1371/journal.pone.0283374)
Supplement: Attachment — Submitted filename: Updated Response to Reviewers.docx [file pone.0283374.s002.docx]

Dear Dr. Hsin-Yen Yen,

We would like to thank you for your consideration of our manuscript *‘Psychosocial health of school-going adolescents during the COVID-19 pandemic: Findings from a nationwide survey in Bangladesh’* and for the feedback provided by the reviewers. We have now addressed each comment provided by the reviewers, and we think the quality of the paper has improved based on the suggestions.

**Response to editor:**

**1. We notice that your manuscript file was uploaded on March 11, 2022. Please can you upload the latest version of your revised manuscript as the main article file, ensuring that does not contain any tracked changes or highlighting. This will be used in the production process if your manuscript is accepted.**

**Please follow this link for more information:** [**http://blogs.PLOS.org/everyone/2011/05/10/how-to-submit-your-revised-manuscript/**](http://blogs.PLOS.org/everyone/2011/05/10/how-to-submit-your-revised-manuscript/)

**Author’s Response**: Thank you for your point. We have uploaded our updated manuscript.

**2. Please provide additional details regarding participant consent. In the ethics statement in the Methods and online submission information, please ensure that you have specified what type you obtained (for instance, written or verbal, and if verbal, how it was documented and witnessed). If your study included minors, state whether you obtained consent from parents or guardians. If the need for consent was waived by the ethics committee, please include this information.**

**Author’s Response**: Thank you for your concern. We obtained e-written consent from the participants and their parents. In the ethics section under methods, we have added this information

Please check page no 11.

**3. You indicated that you had ethical approval for your study. In your Methods section, please ensure you have also stated whether you obtained consent from parents or guardians of the minors included in the study or whether the research ethics committee or IRB specifically waived the need for their consent.**

**Author’s Response**: Thank you for your concern. In our methods section, it was already stated that consent was obtained from parents or guardians of the minors included in the study. Although, the research ethics committee or IRB provided consent for the protocol however, did not waived the need for participants consent.

**Please check page no 11.**

**4. We note that Figures 3 &4 in your submission contain [map/satellite] images which may be copyrighted. All PLOS content is published under the Creative Commons Attribution License (CC BY 4.0), which means that the manuscript, images, and Supporting Information files will be freely available online, and any third party is permitted to access, download, copy, distribute, and use these materials in any way, even commercially, with proper attribution. For these reasons, we cannot publish previously copyrighted maps or satellite images created using proprietary data, such as Google software (Google Maps, Street View, and Earth). For more information, see our copyright guidelines:** [**http://journals.plos.org/plosone/s/licenses-and-copyright**](http://journals.plos.org/plosone/s/licenses-and-copyright)**.**

**We require you to either (1) present written permission from the copyright holder to publish these figures specifically under the CC BY 4.0 license, or (2) remove the figures from your submission:**

**a. You may seek permission from the original copyright holder of Figures 3 &4 to publish the content specifically under the CC BY 4.0 license.**

**We recommend that you contact the original copyright holder with the Content Permission Form (**[**http://journals.plos.org/plosone/s/file?id=7c09/content-permission-form.pdf**](http://journals.plos.org/plosone/s/file?id=7c09/content-permission-form.pdf)**) and the following text:**

**“I request permission for the open-access journal PLOS ONE to publish XXX under the Creative Commons Attribution License (CCAL) CC BY 4.0 (**[**http://creativecommons.org/licenses/by/4.0/**](http://creativecommons.org/licenses/by/4.0/)**). Please be aware that this license allows unrestricted use and distribution, even commercially, by third parties. Please reply and provide explicit written permission to publish XXX under a CC BY license and complete the attached form.”**

**Please upload the completed Content Permission Form or other proof of granted permissions as an "Other" file with your submission.**

**In the figure caption of the copyrighted figure, please include the following text: “Reprinted from [ref] under a CC BY license, with permission from [name of publisher], original copyright [original copyright year].”**

**b. If you are unable to obtain permission from the original copyright holder to publish these figures under the CC BY 4.0 license or if the copyright holder’s requirements are incompatible with the CC BY 4.0 license, please either i) remove the figure or ii) supply a replacement figure that complies with the CC BY 4.0 license. Please check copyright information on all replacement figures and update the figure caption with source information. If applicable, please specify in the figure caption text when a figure is similar but not identical to the original image and is therefore for illustrative purposes only.**

**The following resources for replacing copyrighted map figures may be helpful:**

**USGS National Map Viewer (public domain):** [**http://viewer.nationalmap.gov/viewer/**](http://viewer.nationalmap.gov/viewer/)

**The Gateway to Astronaut Photography of Earth (public domain):** [**http://eol.jsc.nasa.gov/sseop/clickmap/**](http://eol.jsc.nasa.gov/sseop/clickmap/)

**Maps at the CIA (public domain):** [**https://www.cia.gov/library/publications/the-world-factbook/index.html**](https://www.cia.gov/library/publications/the-world-factbook/index.html) **and** [**https://www.cia.gov/library/publications/cia-maps-publications/index.html**](https://www.cia.gov/library/publications/cia-maps-publications/index.html)

**NASA Earth Observatory (public domain):** [**http://earthobservatory.nasa.gov/**](http://earthobservatory.nasa.gov/)

**Landsat:** [**http://landsat.visibleearth.nasa.gov/**](http://landsat.visibleearth.nasa.gov/)

**USGS EROS (Earth Resources Observatory and Science (EROS) Center) (public domain):** [**http://eros.usgs.gov/#**](http://eros.usgs.gov/) **Natural Earth (public domain):** [**http://www.naturalearthdata.com/**](http://www.naturalearthdata.com/)

**We've returned your manuscript to your account. Please resolve these issues and resubmit your manuscript within 21 days. If you need more time, please email the journal office at** [**plosone@plos.org**](mailto:plosone@plos.org)**. We are happy to grant extensions of up to one month past this due date. If we do not hear from you within 21 days, we will withdraw your manuscript.**

**Please log on to PLOS Editorial Manager at** [**https://www.editorialmanager.com/pone/**](https://www.editorialmanager.com/pone/) **to access your manuscript. You will find your manuscript in the 'Submissions Sent Back to Author' link under the New Submissions menu. Be sure to remove your previous manuscript file if you are uploading a new file in response to these requests. After you've made the changes requested above, please be sure to view and approve the revised PDF after rebuilding the PDF to complete the resubmission process.**

**We are requesting these changes to comply with the PLOS ONE submission guidelines (**[**https://journals.plos.org/plosone/s/submission-guidelines**](https://journals.plos.org/plosone/s/submission-guidelines)**). Please note that we won't send your manuscript for review until you have resolved the above requests.**

**Author’s Response**: Thank you for your suggestion. We have removed figure 3 and 4 from our manuscript, which was the map displaying the prevalence of depression and anxiety.

**Responses to Reviewer 1**

**Background**

***Reviewer’s comment:* 1. I am a bit unsure about social change. Can you please explain or term it differently?**

***Authors’ response:*** We have now revised the sentence in the introduction section as follows:

*“Adolescence is an important developmental transition period from childhood to adulthood that includes multiple physical, cognitive and psychosocial changes (1)”*

Please check page no: 5.

***Reviewer’s comment:* 2. I think it would be better if the author could describe the pathways of developing mental health problems in adolescence for pre-covid period and compare it with the situation during COVID-19.**

***Authors’ response:*** Thanks for your suggestion. We have revised the introduction.

*“Depression and anxiety are the most common mental health problems in young and adolescents (2, 3).”*

However, in contrast to the pre-COVID-19 era, additional stressors such as academic delays, the uncertainty of the future, financial crisis, and worry about getting infected were reported for developing mental health issues in adolescents.

Please check page no: 5

***Reviewer’s comment:* 3. Study rationale could be more specifically represented in the study.**

***Authors’ response:*** Thanks for your comment. But this comment did not guide us in any direction. However, we have revised the justification of the study.

*“Several studies conducted with different cohorts including general population, university students, medical students, slum-dwellers, health workers, and COVID-19 survivors highlighted various pandemic related mental health problems (e.g., anxiety, depression, stress, suicidal ideation, and behavioral problems such as problematic use of smartphone,) in Bangladesh (4-15), but research on adolescents during the COVID-19 was very limited at the time of the study.”*

Please check page no: 6

***Reviewer’s comment:* 4. English editing is required**

***Authors’ response:*** We have two native English speakers in the authors’ list. They have now edited our manuscript.

**Methods**

***Reviewer’s comment:* 1. It would be better if the author describe a bit more about eh A2i methodology, that could give the reader a clear sense of it. Although, author have already provided some information about it but its methodology seemed not very clear to me as well as it survey design.**

***Authors’ response:*** We have already added the following paragraph. We have also included a2i’s website (<https://a2i.gov.bd/>) as anyone can browse it to learn about their activity in detail.

*“a2i is a governmental program in the Information and Communication Technology (ICT) Division of Bangladesh that is supported by the Cabinet Division and United Nations Development Programme (UNDP). a2i is a program of the government's Digital Bangladesh agenda which has an edutainment online platform named "Kishore Batayan – Konnect" that has been developed for school-going adolescents [42]. a2i aims to nurture the educational, psychosocial and life skills of school-going adolescents in Bangladesh. Adolescents can share and learn essential life lessons from different creative multimedia content that can help advance their social and personal skills”.*

Please check page no: 7

***Reviewer’s comment:* 2. It will be helpful for the readers to get a sense of why the author had used PHQ-9 and GAD-7 scale in this survey if they describe it briefly.**

***Authors’ response:*** Thanks for your suggestions. We have now added its explanation.

*“Participants’ depression and anxiety were assessed using Patient Health Questionnaire (PHQ-9) and Generalized Anxiety Disorder (GAD-7) measures as these scales were used in several studies in the Bangladesh context including school-going adolescents (16-19) .”*

These scales are also affirmed to be effective and potent screening instruments for anxiety and depression in any demographic as per the previous studies *(16-19)*. Please check page no: 9

***Reviewer’s comment:* 3. How the author has adjusted the potential cofounder and reduce the collinearity of the regression model. Also, what have the author done to adjust the missing values, if there was any?**

***Authors’ response:*** Before performing regression analysis, the potential multi-collinearity was checked by using tolerance (> 0.1) and variance inflation factor (VIF < 10). We had no missing values in the final analysis as we removed all missing data before the final analysis.

Please check page no: 11

***Reviewer’s comment:* 4. Did the author perform any model validity test?**

***Authors’ response:*** We performed the model validity test. We have now included the values of Cox & Snell R Square, and Nagelkerke R Square for each model.

*“The values of Cox & Snell R Square, and Nagelkerke R Square for multivariable logistic regression of depression were 0.27, and 0.36, respectively.”*

*“The values of Cox & Snell R Square, and Nagelkerke R Square for multivariable logistic regression models of depression and anxiety were 0.21, and 0.33, respectively.”*

“*The values of Cox & Snell R Square, and Nagelkerke R Square for multivariable logistic regression models of depression and anxiety were 0.21, and 0.33, respectively*”.

Please check page no:16-18

***Reviewer’s comment:* 5. A brief of categorisation process of explanatory variable would be more helpful as well as measurement process including time duration.**

***Authors’ response:*** We have now added the categorization of explanatory variables in few sections considering the word limit.

Please check page no: 10

***Reviewer’s comment:* 6. Author’s description on outcomes was very clear and specific.**

***Authors’ response:*** Thanks for your positive comments.

***Reviewer’s comment:* 7. In adolesces, behaviours changes with age, so that, I would prefer that it would be better if the author perform age-adjusted prevalence to get the more accurate results.**

***Authors’ response:*** Thanks for your comment. We already adjusted age in each of the multivariable regression analysis. We have added the Figure 3 where the distribution of depression and anxiety with participants’ age is presented.

Please check page no: 16

**Discussion**

***Reviewer’s comment:* It would be better if the author improves the discussion part further, discuss the findings rather repeating the results again. It required substantial revisions and English editing as well to make the findings more understandable**

***Authors’ response:*** Thanks for your suggestion. We have already added both pre-COVID-19 and during COVID-19 studies, and also compared our present findings with the previous studies. We first mentioned the present study findings briefly and then compared our findings with previous studies. However, we have now added some lines to make the discussion more comprehensive as you suggested. We have also edited the English language.

Please check pages no: 21-23, 26-28.

**Responses to Reviewer 2**

***Reviewer’s comment:*** The manuscript was written in standard English. It used appropriate statistical technique considering the dataset leading to a technically sound finding. The conclusions and recommendations made were validly based on the findings. Yes, the data was fully available.

***Authors’ response:*** Thanks for your positive comments. We appreciate your time and review.

**References:**

1.	World Health Organization. Adolescent mental health 2020. 2020.

2.	Merikangas KR, He J-p, Burstein M, Swanson SA, Avenevoli S, Cui L, et al. Lifetime prevalence of mental disorders in US adolescents: results from the National Comorbidity Survey Replication–Adolescent Supplement (NCS-A). Journal of the American Academy of Child & Adolescent Psychiatry. 2010;49(10):980-9.

3.	Racine N, McArthur BA, Cooke JE, Eirich R, Zhu J, Madigan S. Global prevalence of depressive and anxiety symptoms in children and adolescents during COVID-19: a meta-analysis. JAMA pediatrics. 2021;175(11):1142-50.

4.	Hossain MM, Hsan K, Islam MS, Nath SK. Psychological states of Bangladeshi people and associated factors during the outbreak of COVID-19: A cross-sectional survey. Emerging Trends in Drugs, Addictions, and Health. 2021;1:100012.

5.	Islam M, Ferdous M, Islam US, Mosaddek ASM, Potenza MN, Pardhan S. Treatment, persistent symptoms, and depression in people infected with COVID-19 in Bangladesh. International journal of environmental research and public health. 2021;18(4):1453.

6.	Islam MS, Rahman ME, Banik R, Emran MGI, Saiara N, Hossain S, et al. Financial and mental health concerns of impoverished urban-dwelling Bangladeshi people during COVID-19. Frontiers in Psychology. 2021;12:663687.

7.	Islam MS, Sujan MSH, Tasnim R, Mohona RA, Ferdous MZ, Kamruzzaman S, et al. Problematic smartphone and social media use among Bangladeshi college and university students amid COVID-19: the role of psychological well-being and pandemic related factors. Frontiers in psychiatry. 2021;12:647386.

8.	Islam MS, Sujan MSH, Tasnim R, Sikder MT, Potenza MN, Van Os J. Psychological responses during the COVID-19 outbreak among university students in Bangladesh. PloS one. 2020;15(12):e0245083.

9.	Islam M, Tasnim R, Sujan M, Hossain S, Ferdous M, Sikder M, et al. Depressive symptoms associated with COVID-19 preventive practice measures, daily activities in home quarantine and suicidal behaviors: Findings from a large-scale online survey in Bangladesh. BMC psychiatry. 2021;21(1):1-12.

10.	Tasnim R, Sujan M, Hossain S, Islam M, Ritu AH, Siddique M, et al. Prevalence and correlates of anxiety and depression in frontline healthcare workers treating people with COVID-19 in Bangladesh. BMC psychiatry. 2021;21(1):1-14.

11.	Islam M, Rahman M, Banik R, Emran M, Noshin S, Hossain S, et al. Correlates of financial concerns, depression symptoms, and posttraumatic stress disorder symptoms among impoverished urban dwelling individuals in Bangladesh during the COVID-19 pandemic: A face-to-face interview approach. PsyArXiv. 2020.

12.	Islam MS, Ferdous MZ, Potenza MN. Panic and generalized anxiety during the COVID-19 pandemic among Bangladeshi people: An online pilot survey early in the outbreak. Journal of affective disorders. 2020;276:30-7.

13.	Banik R, Islam M, Ahmed M, Koly KN, Mubarak M, Rahman M, et al. General psychiatric symptoms among Bangladeshi people approximately one year after the onset of the COVID-19 pandemic. BMC psychiatry. 2022;22(1):1-15.

14.	Koly KN, Khanam MI, Islam M, Mahmood SS, Hanifi SMA, Reidpath DD, et al. Anxiety and insomnia among urban slum dwellers in Bangladesh: the role of COVID-19 and its associated factors. Frontiers in Psychiatry. 2021:2115.

15.	Hasan MT, Hossain S, Safa F, Anjum A, Khan AH, Koly KN, et al. Anxiety and depressive symptoms among physicians during the COVID-19 pandemic in Bangladesh: a cross-sectional study. Global mental Health. 2022;9:285-97.

16.	Anjum A, Hossain S, Sikder T, Uddin ME, Rahim DA. Investigating the prevalence of and factors associated with depressive symptoms among urban and semi-urban school adolescents in Bangladesh: a pilot study. International health. 2022;14(4):354-62.

17.	Islam MS, Rahman ME, Moonajilin MS, van Os J. Prevalence of depression, anxiety and associated factors among school going adolescents in Bangladesh: Findings from a cross-sectional study. PLoS One. 2021;16(4):e0247898.

18.	Saiful Islam M, Rahman ME, Moonajilin MS, Griffiths MD. Validation and evaluation of the psychometric properties of Bangla nine-item Internet Disorder Scale–Short Form. Journal of Addictive Diseases. 2020;38(4):540-9.

19.	Moonajilin MS, Rahman ME, Islam MS. Relationship between overweight/obesity and mental health disorders among Bangladeshi adolescents: a cross-sectional survey. Obesity Medicine. 2020;18:100216.
